# Hyperdynamic microtubule-based structural changes in Monocyte-Derived-Neuronal-like cells from patients with schizophrenia

**DOI:** 10.1038/s41537-026-00751-0

**Published:** 2026-04-30

**Authors:** Alfredo Bellon, Alonso Cortez-Resendiz, Lauren N. Forrest, Omar Elmarasi, Janani Iyer, Anjali Iyer, Chachrit Khunsriraksakul, Dajiang Liu, L. Elliot Hong, Therese M. Jay, Marie-Odile Krebs, Anne Hosmalin

**Affiliations:** 1 Penn State Hershey Medical Center, Department of Psychiatry and Behavioral Health, Hershey, PA USA; 2 Penn State Hershey Medical Center, Department of Pharmacology, Hershey, PA USA; 3 Saint Louis University School of Medicine, St. Louis, MO USA; 4 University of Michigan, Ann Arbor, MI USA; 5 Penn State University College of Medicine, Hershey, PA USA; 6 Department of Public Health Sciences; Penn State University College of Medicine, Hershey, PA USA; 7 Department of Psychiatry and Behavioral Sciences at McGovern Medical School, The University of Texas Health Science Center at Houston, Houston, TX USA; 8 Université Paris Cité, Institute of Psychiatry and Neuroscience of Paris (IPNP), INSERM U1266, Pathophysiology of Psychiatric Disorders, Paris, France; 9 Université Paris Cité, Institute of Psychiatry and Neuroscience of Paris (IPNP), INSERM U1266, Pathophysiology of psychiatric disorders, GDR 3557-Institut de Psychiatrie, Paris, France; 10 GHU Paris Psychiatrie et Neurosciences, Pôle Hospitalo-Universitaire d’évaluation, Prévention, et Innovation Thérapeutique (PEPIT), Paris, France; 11 Université de Paris, Institut Cochin, CNRS, INSERM, Paris, France

**Keywords:** Schizophrenia, Cellular neuroscience

## Abstract

A consistent postmortem finding in schizophrenia (SCZ) is reduction in dendrites’ size. However, neurons with larger dendritic trees have also been encountered. In vitro experiments with neurons and neuronal-like cells coming directly from patients with SCZ showed similar results, evidencing at times more extensions and at others less arborizations. The process of extending and retracting neuronal outgrowths depends on microtubules polymerization and depolymerization. The possibility that microtubule polymerization/depolymerization is related to defects in the neuronal structure comes from several microtubular anomalies reported in SCZ such as its abnormal distribution in the cytoplasm, irregular shape of microtubules and even absence of these cytoskeletal components in dendrites. Moreover, microtubules in olfactory neuroepithelial cells from patients with SCZ were resistant to depolymerization. But whether deficits in microtubules are associated with abnormalities in the neuronal structure has never been investigated in living cells coming directly from patients. Therefore, we studied dynamic neurostructural changes in Monocyte-Derived-Neuronal-like cells (MDNCs) from 12 controls and 13 patients with SCZ. First, we showed that human neuroprogenitor cells and MDNCs have comparable neurostructural plasticity. Then, we investigated whether colchicine, a microtubular stabilizing and depolymerizing agent, disrupts dynamic neurostructural changes. The lowest concentration of colchicine tested, stopped dynamic neurostructural changes in MDNCs from controls, while cells from patients with SCZ continued to extend and retract neuronal outgrowths. Following, we investigated if antipsychotics impact dynamic structural changes, but our results were inconclusive. Our data indicate that, under certain circumstances, neuronal-like cells from patients with SCZ evidenced hyperdynamic microtubule-based neurostructural changes and consequently, link deficits in microtubules with anomalies in the neuronal shape. While other potential confounders are unlikely to have influenced our results, the effects of medications cannot be excluded.

## Introduction

The neurodevelopmental hypothesis of schizophrenia (SCZ) remains the most comprehensive theory to explain this illness^1–4^. Such hypothesis was first developed based on clinical and epidemiological data indicating that complications during pregnancy like preeclampsia, low gestational age and low birth weight double the risk for SCZ^2^. Minor physical anomalies, especially those involving the face and cranium are more commonly found in SCZ than non-affected individuals, which also suggests this illness originates during early brain development^2^. More recently, genetic studies^5–7^ and work with stem cells^8^ are accumulating data pointing to early neurodevelopment as a critical period in the pathophysiology of this psychotic disorder. For instance, genome-wide association studies (GWAS) indicate that many genes associated with SCZ are involved in early stages of brain development^5–7^ and some of these genes impact cortical size later in life^9^. Copy number variants (CNVs), which are structural variations in the genome, have also been associated with changes in cortical size. A current study found that increased CNVs penetrance was associated with lower cortical size in SCZ^10^. The CNVs included in this report were those that increase risk for neurodevelopmental disorders^10^. Another line of evidence that supports the neurodevelopmental hypothesis of SCZ is data emerging from Induced Pluripotent Stem Cells (IPSCs). IPSCs primarily model prenatal neurodevelopment stages^11^ including the production and differentiation of neuroprogenitor cells (NPCs). IPSCs-derived NPCs from patients with SCZ have shown impaired cell cycle control^12^, while SCZ forebrain organoids, evidenced a higher number of NPCs when compared to control organoids^13^. But despite the mounting evidence suggesting early development is implicated in the pathophysiology of SCZ, which developmental process is at fault remains unclear. Appraising postmortem results as an entire body of evidence, instead of one study at a time, while complementing it with recent data originating from stem cells and other cellular methodologies, leads us to propose that the capacity to modify the neuronal structure may be compromised in SCZ.

Neurons experience drastic structural changes to evolve from a rounded neuroblast into a ramified neuron. This transformation rarely consists of continuous outgrowth of neuronal extensions. Instead, it is characterized by extension and retraction of neurites^14,15^. Neurites, which are immature neuronal outgrowths, are highly dynamic^14^. As development progresses and neurites mature into either dendrites or axons, the constant cycle of outgrowth and retraction becomes less evident, but it nonetheless persists^16–20^. Such back and forth movements appear to be a mechanism to explore the extracellular environment to guide dendrites and axons to their correct connecting target^15^. Through these connections, dendrites and axons first organize into local circuits that continue to be refined and are later expanded into long-range unified networks projecting throughout the entire brain^21^. Thus, neurite extension and retraction occurring early in development is essential for normal brain development. Moreover, dynamic changes in the neuronal structure do not stop with the establishment of mature brain circuits. In fact, extension and retraction of neuronal arborizations is still observed in the adult brain. But in adulthood, these progressive and regressive structural steps are limited to branches and distal ends of axons^22–24^ and dendrites^19,25,26^. Retaining some degree of structural plasticity in adulthood is evident even in the absence of any environmental stimuli^22–25,27–29^ and becomes more pronounced during learning^30,31^ and in periods of prolonged stress^32,33^.

The neuronal structure is established by rearrangements in microtubules and microfilaments; two main components of the cytoskeleton^14,34,35^. Microtubules’ essential role in sculpting the neuronal shape consists in alternating between bouts of polymerization (growth) and depolymerization (retraction)^14,34,36,37^. Alternating between polymerization and depolymerization depends on the assembly and disassembly of tubulin; the protein that forms microtubules^35,37^. Colchicine is one of several pharmacological compounds that can disrupt tubulin polymerization. At low concentrations, colchicine binds to tubulin and this tubulin-colchicine complex halts growth of microtubules^38–41^. With microtubule dynamics arrested, the neuronal shape remains static^38^. At higher concentrations however, colchicine triggers microtubule depolymerization^41^ and consequently, leads to neurite retraction^41–44^. Another pharmacological compound that can disrupt microtubule dynamics is nocodazole. But in contrast to colchicine, nocodazole exerts both effects on microtubules, independently of its concentration^45^. Nocodazole has been employed to study microtubules in SCZ using olfactory neuroepithelial cells (ONCs). The olfactory epithelium’s constant state of regeneration provides a source of neurons that covers numerous developmental stages going from neuroprogenitor cells to mature olfactory sensory neurons^46,47^. Access to these stratified stages of neuronal differentiation is, for some authors, a potential window into human neurodevelopment^46^. Hence, the use of ONCs in SCZ. Brown and colleagues found that microtubules in ONCs from patients with SCZ did not respond comparably to microtubules in cells from healthy controls^48^. Nocodazole, led to depolymerization of microtubules in control cells, while in patients’ cells, microtubules were only minimally affected^48^. Thus, microtubules in ONCs from patients with SCZ, were resistant to the depolymerization effects of nocodazole (Supplementary Table 1). Other anomalies in microtubules have also been reported. A different research team found that olfactory neuronal cells from patients with SCZ lacked the normal cytoplasmic distribution of microtubules seen in cells from healthy controls^49^. Since these experiments were conducted via immunofluorescence against β-III-tubulin, diminished expression of this protein could be a confounder. However, western blots revealed no differences in the amount of β-III-tubulin between olfactory neuronal cells from SCZ and cells from controls^49^. Therefore, the authors concluded that their findings were consistent with microtubule abnormalities and not deficiencies in β-III-tubulin expression^49^. In line with these results is a study reporting unchanged expression of β-tubulin in postmortem brains from individuals with SCZ and controls^50^. But contrasting results also coming from postmortem brains have been published. Western blots revealed decreased expression of β-I-tubulin in one brain area and increased in another brain region^51^, while proteomics showed lower levels of β^52,53^and α-tubulin^53^ (Supplementary Table 1). Whether tubulin expression is increased or decreased in SCZ remains to be elucidated, but genetic analyses based on genome-wide association results suggest that microtubule-based processes are linked to SCZ^54^ while a comparison between different brain illnesses indicates SCZ has the second highest proportional association of microtubule-related genes just after amyotrophic lateral sclerosis^55^.

Comparable to findings from olfactory neuroepithelial cells, postmortem assessments at the ultrastructural level also evidenced less or even absent microtubules in dendrites from the prefrontal and limbic cortex of patients with SCZ^56–58^ (Supplementary Table 1). Interestingly, brain cells from embryos obtained from women with SCZ undergoing medical abortions showed instead, a higher number of microtubules when compared to control cells^59^. In addition, these microtubules lacked its normal orientation and its shape was convoluted^59^. Considering microtubules play an essential role in establishing the neuronal shape, the possibility exists that deficits in microtubule dynamics would lead to abnormalities in the neuronal structure in SCZ. While this causal effect has not been investigated, deficits in the neuronal structure have been consistently reported. One of the most common neurostructural defects in SCZ is shrinkage in neuropil characterized by decreased dendritic spines^60–62^, reductions in dendritic length^63–65^ and lower number of dendritic branches^66,67^. But the opposite has also been found. An increased number of dendrites^68^ and axons^69^ has been encountered in postmortem brains from patients with SCZ, while documentation of neurons with abnormal shape^70^ and malformed neurites^71,72^ also exists. Cellular approaches to SCZ showed similar results. Maturing neurons generated from patients’ pluripotent cells not yet committed into any particular neuronal type evidenced longer neuronal extensions very early in development^73,74^, whereas shorter neuronal outgrowths are present in more advanced stages of differentiation^75–77^. This combination of findings showing at times more extensions and at others less arborizations could potentially be explained by deficits in the process of extending and retracting neuronal outgrowths. To study these dynamic structural changes directly in living cells from patients with SCZ, we utilized Monocyte-Derived-Neuronal-like cells (MDNCs).

We developed a protocol to transdifferentiate human circulating monocytes into neuronal-like cells in 20 days and without the need for reprogramming^78^. These MDNCs conduct electrical activity and express numerous neuronal markers concomitantly with a significant drop or complete absence in the expression of genes and proteins present exclusively in monocytes^78,79^. Other cellular approaches to SCZ have been criticized by its lack of reproducibility^80–85^. We have shown that MDNCs deliver reproducible results in several neurostructural parameters^74,78^. We have also evidenced that the structure of MDNCs is comparable to that of Human Developing Neurons (HDNs) kept in culture for 5 days^78^. Furthermore, treatments with colchicine led to similar changes in neuronal extensions of MDNCs and HDNs^74^. Given the similarities between MDNCs and HDNs, we recently compared MDNCs from patients with SCZ to cells from unaffected individuals. MDNCs from patients with SCZ evidenced a higher percentage of differentiation and a more complex structure^74^. In addition, dopamine elicited increased pruning of primary neurites in a subgroup of patients^74^. This subgroup of patients also revealed lower expression of dopamine 1 receptors^74^. Considering that MDNCs deliver reproducible results, develop a neurostructure comparable to that of HDNs, respond to colchicine similarly than HDNs and can be generated in 20 days, we chose MDNCs as our experimental approach.

Since several lines of evidence indicate neurons from patient with SCZ present impairments in microtubules^48,49,56–58^, while other studies have consistently shown abnormalities in the neuronal shape^63–67^, we hypothesize that neurons from patients with SCZ will evidence anomalies in dynamic neurostructural changes which consist of constant extension and retraction of arborizations and that those neurostructural deficiencies will be driven by microtubules. To test this hypothesis, we first studied if the structural dynamism present in MDNCs is comparable to that of human neuroprogenitor cells (NPCs) or that of HDNs. We then examined if MDNCs from patients with SCZ present deficits in dynamic neurostructural changes at baseline; meaning, under control culture conditions. Following, we studied whether colchicine halts modifications in the neurostructure of cells from patients with SCZ at the same rate as in control cells. Finally, we investigated whether antipsychotics influenced our results.

## Materials and Methods

### Subjects

Participants gave their informed and written consent after receiving full description of the study. Experiments pertaining to the cohort of patients and controls were approved by the ethics committee Ile de France II, while experiments on MDNCs involving only control individuals were approved by the Institutional Review Board at Penn State University (Study #00006911). Patients were diagnosed using the Diagnostic and Statistical Manual of Mental Disorders, 4^th^ Edition (DSM-IV) criteria. Diagnosis was based on clinical interviews and medical records. Patients were recruited from the Department of Psychiatry at Sainte-Anne Hospital in Paris, France. Healthy controls were recruited through local advertisements. Controls were screened to rule out any past or present history of DSM-IV axis 1 disorders. Only individuals older than 18 years old were recruited.

Thirty-five subjects, 16 controls and 19 patients with SCZ, were recruited for this study. In the SCZ group one patient was diagnosed with schizoaffective disorder and another with pervasive developmental disorder and psychosis while one control had hemochromatosis. One patient and one control provided blood samples twice. The second blood draw was obtained at least two months after the original sample was obtained. Thus, these two individuals were included as duplicates in the statistical analysis. Five patients and three controls were excluded from the study due to mistaken concentrations of growth factors used for transdifferentiation. Twelve controls and 13 patients were included in the statistical analysis. Of the 13 patients included, two were unmedicated. A different analysis from this cohort of patients and controls was presented in a prior publication where we provided detailed information about all the individuals included in this study^74^. Succinct data about patients and controls including gender, age and number of differentiated cells is listed in Table 1 while sample sizes for all analyses are included in Table 2. The number of MDNCs each individual contributed per experiment comparing patients versus controls is described in Supplementary Table 2.

**Table 1:** Gender, age and cell differentiation from control individuals versus patients with schizophrenia.

	Controls (*n* = 13^a^)	Schizophrenia (*n* = 14^b^)	*p*
Gender	77% men	86% men	*p* = 0.64
Age^c^ (range)	32.6 ± 3.2 (19–65 years)	33.2 ± 3.4 (19–67 years)	*p* = 0.88
Number of differentiated cells^c^	6.70 ± 0.95 (*n* = 3933)	8.07 ± 0.90 (*n* = 6144)	*p* = 0.31

^a^One individual was tested twice.

^b^One patient was tested twice.

^c^mean ± SEM.

**Table 2:** Sample sizes for SDI analyses.

	CTL	SCZ	MED	UNMED^*^
Control				
Subjects	8	10	8	2
MDNCs	465	879	732	147
Colchicine 0.4				
Subjects	4	7	6	1
MDNCs	266	379	363	16
Colchicine 0.5				
Subjects	4	11	10	1
MDNCs	397	932	896	36
Colchicine 0.75				
Subjects	3	9	8	1
MDNCs	296	809	780	29

*CTL* control individuals, *SCZ* patients with schizophrenia, *MED* medicated patients with SCZ, *UNMED* unmedicated patients with SCZ.^*^Note, for conditions where there was only 1 participant in the SCZ-unmedicated group, the CTL vs. SCZ-unmedicated comparisons could not be performed.

### Cell culture

We followed our transdifferentiation protocol which has been published and described in detail elsewhere^78^. Briefly, monocytes were isolated from blood using CD14+ magnetic microbeads (Miltenyi Biotec, 130-050-201). A fraction of peripheral blood mononuclear cells (PBMCs) was cultured in parallel. Both, monocytes and PBMCs were cultured on human fibronectin (Sigma-Aldrich, F2006; 20 µg/ml) coated plates or flasks. Macrophage colony-stimulating factor (AbCys, 300-25; 50 ng/ml) was added to monocytes right before culturing. Cells were maintained in Dulbecco’s Modified Eagle Medium (DMEM), High Glucose, GlutaMAX (GIBCO, 61965059) in which we added 100 U/mL penicillin; 100 mg/mL streptomycin, 1% nonessential amino acids, 1 mM sodium pyruvate, 10 mM HEPES buffer, (all from Life Technologies) and supplemented with 10% fetal bovine serum from GIBCO Performance Plus. Media was changed on days 4, 7, 10 and 13. On day 4, new DMEM and PBMC-conditioned media at a rate of 2:1 was used. For days 7, 10 and 13 the DMEM/PBMC-conditioned media rate used was 1:1. On day 7, Butylated hydroxyanisole (BHA) (Sigma-Aldrich, B1253) was included at a final concentration of 50 nM. On day 10, retinoic acid (RA) (Sigma-Aldrich, R2625) at a final concentration of 16 µM was added to BHA 50 nM. On day 13, BHA 50 µM, RA 16 µM, Insulin Growth Factor-1, 12.5 ng/ml (Peprotech, 100-11) and Neurotrophin-3, 30 ng/ml (Peprotech, 450-03-100) were applied. Potassium chloride 25 mM (Sigma-Aldrich, P5405) was added to culture plates on day 17 without changing the culture media. Neuronal-like cells become apparent on or after day 20^78^. Treatments with colchicine (Sigma-Aldrich, C9754) involved three concentrations; 0.4 µM, 0.5 µM and 0.75 µM. Incubation time was 1 h, just as what has been used in other neuronal-like cells^42,43^. All analyses were done blinded.

Human neurons were obtained from ScienCell Research Laboratories (1520-10) and cultured following the manufacturer’s instructions. Pictures of human neurons were taken after 5–7 days in culture. Human neuroprogenitor cells from Lonza (PT-2599) were cultured according to the manufacturer’s recommendations using Neuroprogenitor Maintenance Medium (Lonza, CC-3209 plus CC-4241 & CC-4242). However, we enriched maintenance medium with Leukemia Inhibitory Factor (Millipore, LIF1050) at 10 ng/ml 24 h after culture similarly the protocol followed by Carpenter et al.^86^. Neuroprogenitor cells were maintained with weekly media change for 47 days when differentiation was launched. For differentiation, we followed the manufacturer’s instructions and used their differentiation media (CC-3229 plus CC-4242) but we supplemented such media with Neurotrophin-3 at 30 ng/ml (Peprotech, 450-03-100). Pictures of neuroprogenitor cells were taken on days 54 and 55 of culture.

Pictures of all cells were taken using a Nikon Eclipse Ti-S/L 100 inverted microscope equipped with a CoolSNAP Myo, 20 MHz, 2.8 Megapixel, 4.54 × 4.54 µm pixels camera and with a Nikon CFI Super fluor 20X DIC prism objective.

### Structural dynamic index (SDI)

**Fig. 1:**
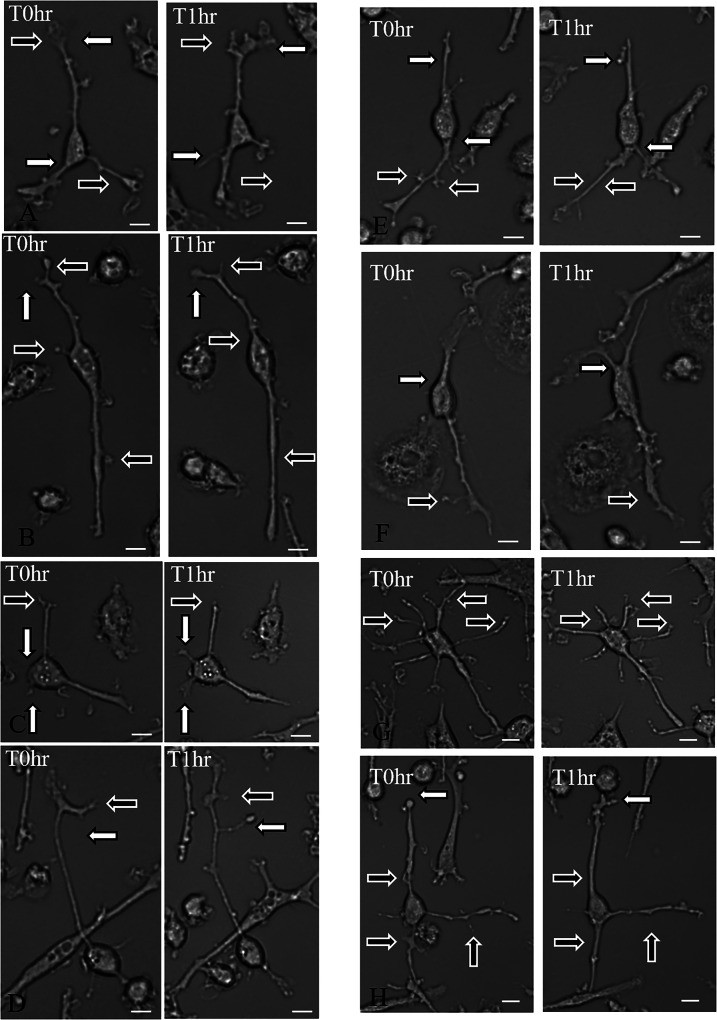
Light microscopy pictures of Monocyte-Derived-Neuronal-like cells (MDNCs). Each MDNC was photographed at time zero (T0hr) and after 1 h of incubation (T1hr) (20x original magnification). White arrows point to growth of neurites, while black arrows identify retraction of neuronal extensions. **A** Microscopy pictures of an MDNC from a healthy control. **B**, **C** Microscopy pictures of MDNCs from a different healthy individual. **D** Microscopy pictures of an MDNC from yet another healthy control. **E**, **F** Microscopy pictures of MDNCs from a patient with schizophrenia. **G** Microscopy pictures of an MDNC from a different patient with schizophrenia. **H** Microscopy pictures of an MDNC from yet another patient with schizophrenia. Scale bar = 20 µm.

To determine neurostructural changes, light microscopy photographs of the exact same cells were taken at time zero (T0hr) and then after 1 h of incubation (T1hr) either under control conditions (Fig. 1) or after treatment with three different concentrations of colchicine. Identification of cells was achieved via micro-ruled coverslips (Cellattice CLS5-25D, Nexcelom Bioscience, Lawrence, MA, USA). Having pictures of the exact same cells at T0hr and then at T1hr allowed us to compare whether MDNCs had, within an hour, undergone any structural transformation consisting of either growth or retraction of neurites (Fig. 1). Often, neurostructural reports concentrate on either retraction or extension of neuronal outgrowths but our approach, allowed us to include both at the same time. Each structural change was assigned a score as described in Table 3. The more drastic the structural change, the higher the score. For instance, either complete retraction or outgrowth of a neurite larger than two times the soma size, received six points. In contrast, total retraction or outgrowth of a tertiary neurite, received only one point, as tertiary neurites tend to be very short in length and therefore considered a less drastic neurostructural transformation than the loss or outgrowth of a primary neurite larger than two times the soma size. The sum of all scores was considered the Structural Dynamic Index (SDI). We relied on light microscopy photographs to avoid staining methods which are known to alter the neuronal structure^87–89^. There are automated methods that can measure dynamic structural changes in neurons and neuronal-like cells, however automated methods at times, fail to account for segments of neurites as we presented in a prior publication^79^. Even other researchers promoting real-time analysis of live neuronal cultures^90^ or testing machine learning algorithms^91^ acknowledged that manual methods continue to be the gold standard to study dynamic changes in the neuronal structure. Therefore, our analysis was performed via manual counting of structural rearrangements and scored based on the instructions described on Table 3. All our analyses were performed blinded.

**Table 3:** Scoring rubric used to quantify structural changes in each cell.

Neurite category	Structural change	Points
Primary neurite ≥2x soma size	Completely lost or created	6
Primary neurite ≥2x soma size	Decreased to <2x soma size	5
Primary neurite <2x soma size	Increased to ≥2x soma size	
Primary neurite <2x soma size	Completely lost or created	4
Primary neurite ≥2x soma size	Decreased or increased in size but remains ≥ 2x soma size	3
Primary neurite <2x soma size	Decreased or increased in size but remains < 2x soma size	
Secondary neurite >2x soma size	Completely lost or created	
Secondary neurite >2x soma size	Decreased to ≤2x soma size	2
Secondary neurite ≤2x soma size	Completely lost or created	
Secondary neurite ≤2x soma size	Decreased or increased in size	
Tertiary neurite	Completely lost or created	1
Quaternary neurite	Completely lost or created	0.5

### Single-cell RNA sequencing

We performed a transcriptome-wide single-cell mRNA sequencing of 17 monocytes exposed to our transdifferentiation protocol as described previously^78^. These 17 cells were selected based on size. Only medium-size cells were selected for single-cell mRNA sequencing as small cells are typically monocytes, and large cells often correspond to macrophages. The soma size targeted was between 20 to 30 µm. Briefly, single-cell capture by Fluidigm’s C1^TM^ Single-Cell Autoprep System was obtained through an integrated fluidic circuit chip. After optical confirmation of cell number at each capture site on the chip, the cells were processed for in-line cell lysis, reverse transcription and cDNA amplification. The resulting cDNA was subjected to a sequencing library using Illumina’s Nextera XT library preparation kit. The Rapid mode of Illumina HiSeq 2500 was used to generate sequencing reads of sufficient depth (about 3 million of sequencing reads) per cell. De-multiplexed sequencing reads passed the default quality filtering of Illumina CASAVA pipeline (v1.8) and were then exposed to further quality trimmed/filtered using FASTX-Toolkit (v.0.0.13). The filtered reads were aligned to the reference genome (hg38) using Tophat (v2.0.9)^92^. After normalization was performed via the median of the geometric means of fragment counts across all libraries, Fragments Per Kilobase per Million mapped reads values were calculated using Cuffdiff tool which is available in Cufflinks version 2.2.1^93^. In addition, we quantified library complexity and expression coverage for the 17 monocyte-derived neuronal cells used in the analysis. For each cell, we computed (1) total reads per cell and (2) number of detected genes (count > 0). All 17 cells showed high-quality profiles:

(1) Mean reads per cell: 5.3 × 10⁶, Range: 2.4 × 10⁶ – 6.9 × 10⁶ (2) Mean genes per cell: 7600, Range: 5600–9400

No cells exhibited low library complexity or unusually low gene detection, supporting inclusion of all 17 cells in downstream analyses. To visualize cell-to-cell variability, we generated a heatmap using the representative marker genes across the 17 selected cells (Supplementary Fig. 1).

### Scmap

We used scmap as described by Kiselev at el.^94^. To annotate each query profile by comparison to a labeled reference single-cell RNA sequencing dataset. After harmonizing gene identifiers between reference and query (restricting to overlapping genes and removing duplicates), we selected informative genes from the reference using “*selectFeatures”*, and built a reference index by summarizing each labeled reference group into a representative centroid expression profile using *“indexCluster”*. We then projected each query profile onto the reference index with *“scmapCluster”*, assigning it to the best-matching reference group based on similarity; profiles were labeled unassigned if the maximum similarity did not exceed the scmap threshold of 0.7, as described by Kiselev et al.^94^. As a sensitivity analysis, we repeated the mapping using 50 vs. 100 selected features (n_features = 50 and n_features = 100) (Supplementary Fig. 2).

### Statistical analysis

To compare patients and controls, we first averaged picture-level data for each sample and then averaged sample-level data within each subject to calculate descriptive statistics and effect sizes. Following, we performed a series of multilevel models to determine if there were differences in SDI in MDNCs within each condition, between those individuals with SCZ and CTL. All models included random intercepts to account for the nested observations (cells nested within samples, samples nested within participants). Test statistics and 95% confidence intervals were estimated with *n* = 1000 bootstrap resampling, given that SDI was not normally distributed within groups or conditions. These analyses were performed in R using the lme4 package. One-way ANOVA with Tukey post hoc tests were used to study the effects of haloperidol and clozapine on MDNCs. The Kolmogorov-Smirnov Test of Normality was used to assess if data included in correlation analyses were normally distributed. *P* values lower than 0.05 and 95% confidence intervals that did not span 0 were considered significant.

### Experimental design

Based on the criteria described above, we determined the SDI for human neuroprogenitor cells (NPCs) and human developing neurons (HDNs). Then, to establish where the neurostructural plasticity of MDNCs falls in the neurodevelopmental continuum between NPCs and HDNs, we quantified the structural dynamic index for MDNCs and compared it with that of human neuroprogenitor cells and human developing neurons. To determine if our SDI results were supported by genetic data, we performed single-cell RNA sequencing of MDNCs and compared it to previously published single-cell RNA sequencing done with NPCs and HDNs. Once established whether MDNCs resemble either NPCs or HDNs genetically and neurostructurally, we then compared the SDI of MDNCs from patients with SCZ versus cells from control individuals. Comparisons between cells from patients versus controls were done after four different culture conditions. First, SDI was calculated from MDNCs cultured under control conditions. Then, MDNCs were exposed in parallel to three different concentrations of colchicine: 0.4, 0.5 and 0.75 µM. These concentrations were selected because they are known to comparably affect the neurostructure of MDNCs^78^, neurons^38^, and neuronal cell lines^95^. Finally, to determine the potential influence of antipsychotics on our results, MDNCs were cultured in parallel with circulating concentrations of haloperidol (20 ng/ml) and clozapine (500 ng/ml) using the vehicle (DMSO) as control. These incubations were conducted from day 4 to 7 of the transdifferentiation process, which resembles the circumstances in which monocytes in circulation are exposed to antipsychotics in living patients. These monocytes treated with antipsychotics were then exposed to either colchicine 0.4 µM or the vehicle once they were transdifferentiated into MDNCs. Then the SDI of these MDNCs was calculated and compared between groups.

## Ethics approval and consent to participate

Participants gave their informed and written consent after receiving full description of the study. Experiments pertaining to the cohort of patients and controls were approved by the ethics committee Ile de France II, while experiments on MDNCs involving only control individuals were approved by the Institutional Review Board at Penn State University (Study #00006911).

## Results

### Structural dynamic changes in human neuroprogenitor cells, human developing neurons and MDNCs

**Fig. 2:**
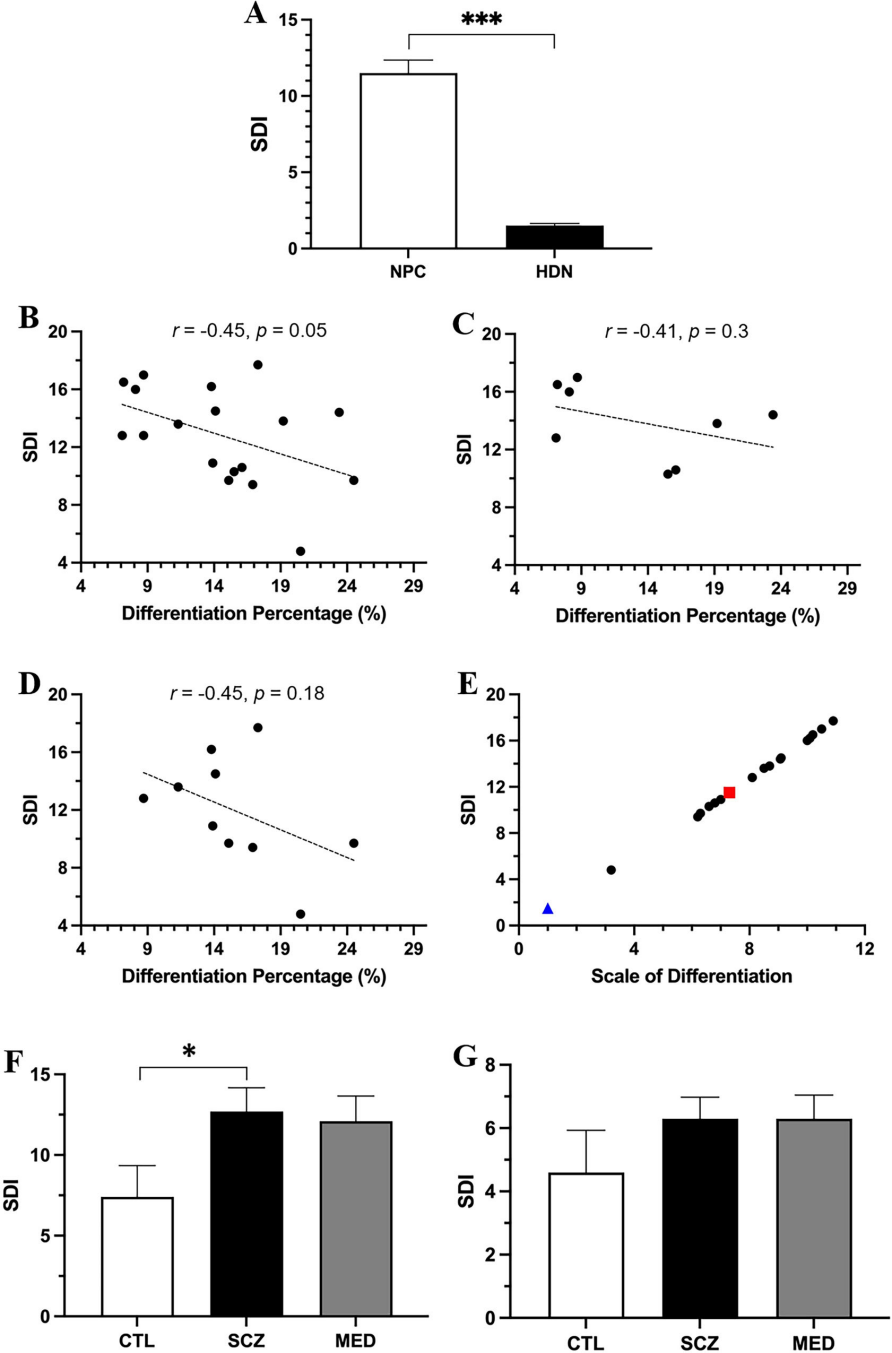
Structural Dynamic differences in human developing neurons, human neuroprogenitor cells and MDNCs. **A** Difference in Structural Dynamic Index (SDI) between human neuroprogenitor cells (NPCs) and human developing neurons (HDNs), ***p < 0.00001. Data is presented as mean ± SEM. **B** Correlation between differentiation percentage and SDI in MDNCs from all 18 subjects. **C** Correlation between differentiation percentage and SDI in MDNCs from 8 control individuals. **D** Correlation between differentiation percentage and SDI in MDNCs from 10 Patients with schizophrenia. **E** Linear graph that shows MDNCs closer to NPCs than to HDNs based on level of differentiation and SDI. NPCs are represented by the red square, while the blue triangle denotes HDNs. **F** Bar graphs comparing SDI between MDNCs from control individuals (CTL; *n* = 266), patients with schizophrenia (SCZ; *n* = 379) and medicated patients (MED; *n* = 363) after treatment with colchicine 0.4 µM, *(*b* = 5.27, 95% CI [0.64, 10.05]). **G** Bar graphs comparing SDI between MDNCs from CTL (*n* = 397), SCZ (*n* = 932) and MED (*n* = 896) after treatment with colchicine 0.5 µM. For **F** & **G**, data is presented as mean ± SEM.

In vitro and in vivo studies in rodents^19,31^ as well as indirect evidence from humans^96,97^ have shown that as brain development progresses, neurons’ structural plasticity diminishes. To test whether this is true for human neurons in vitro at two different stages of development, we contrasted NPCs and HDNs. We measured neurostructural changes in vitro and generated an SDI as described in the methods section. As expected, NPCs had a significantly higher SDI than HDNs (NPCs 11.5 ± 0.86; HDNs 1.5 ± 0.15; *p* < 0.00001) (Fig. 2A) as NPCs are not as differentiated as HDNs. To determine if MDNCs follow this same progression, we investigated in the 18 subjects from whom we obtained pictures at T0hr and T1hr, the correlation between differentiation percentage and SDI. We found a statistically significant negative correlation between these two variables (*r*(16) = −0.45, *p* = 0.05), suggesting that, similarly to NPCs and HDNs, in MDNCs as differentiation advances, dynamic neurostructural changes decrease (Fig. 2B). Since these 18 subjects include patients with SCZ and controls (CTL), we performed correlations with each cohort separately and results were comparable (Fig. 2C, D). All these analyses were done blinded.

NPCs and HDNs represent two different stages of neuronal development, and both cell types showed contrasting SDI scores. To determine whether dynamic structural changes in MDNCs are closer to either HDNs or NPCs, we created a scale of differentiation based on SDI. HDNs are the most differentiated cells in our scale, thus their SDI score of 1.5 (Fig. 2A) was assigned a differentiation scale of 1 (Fig. 2E). With this premise as starting point, and based on proportionality, we calculated the differentiation score for NPCs and for MDNCs from 18 subjects. As shown in Fig. 2E, MDNCs from one individual were closer to HDNs, while MDNCs from all other subjects were closer to NPCs. We then assessed whether transcriptomic profiles support our SDI results. We compared via scmap^94^, single-cell mRNA sequencing data from 17 MDNCs to transcriptomic profiles of developing human cortex. These profiles obtained by Pollen et al., identified signature genes for NPCs (NPCs; *n* = 15 cells), HDNs from gestational week 16 (GW16; *n* = 26 cells), HDNs from gestational week 21 (GW21; *n* = 8 cells), and HDNs from GW21 kept in culture for an additional 3 weeks to further its differentiation (GW21 + 3; *n* = 16 cells)^98^. Using 50 signature genes (Supplementary Table 3), scmap identified 12 out of 17 MDNCs as human NPCs while two MDNCs were grouped with HDNs at GW21 and three cells were unassigned (Table 4 & Supplementary Fig. 1). If one hundred signature genes are utilized (Supplementary Table 3), results remain similar (Supplementary Table 4). In order to determine, also via scmap whether MDNCs retain a monocytic lineage, genes specific for monocytes were used (Supplementary Table 5), all 17 MDNCs were unassigned regardless of whether 50 (Table 5) or 100 genes were employed (Supplementary Table 6 & Supplementary Fig. 2).

**Table 4:** Characterization via scmap of MDNCs based on 50 Human Developing Cortex genes.

Mdnc	Cluster	Score
1	NPC^a^	0.782022272
2	NPC	0.768774524
3	NPC	0.821988117
4	unassigned	0.177159873
5	NPC	0.821299096
6	unassigned	0.695147665
7	NPC	0.748643732
8	NPC	0.817859461
9	NPC	0.781660132
10	GW21^b^	0.712793825
11	NPC	0.807119435
12	GW21	0.780702156
13	unassigned	0.634693623
14	NPC	0.834619246
15	NPC	0.844910537
16	NPC	0.703033654
17	NPC	0.810002581

^a^Neuroprogenitor cell.

^b^Gestation week 21.

**Table 5:** Characterization via scmap of MDNCs based on 50 monocyte-specific genes.

Mdnc	Cluster	Score
1	unassigned	0.319475197
2	unassigned	0.320962085
3	unassigned	0.316000672
4	unassigned	0.151394159
5	unassigned	0.398256092
6	unassigned	0.311483449
7	unassigned	0.258188576
8	unassigned	0.219829784
9	unassigned	0.292014882
10	unassigned	0.19989427
11	unassigned	0.227595498
12	unassigned	0.381237117
13	unassigned	0.198273711
14	unassigned	0.341830569
15	unassigned	0.28354892
16	unassigned	0.243233163
17	unassigned	0.298526572

### Structural dynamic changes in MDNCs from individuals with SCZ and CTL

On a recent publication we reported that MDNCs from individuals with SCZ and CTL did not show differences in pruning of neurites after treatment with three different concentrations of colchicine (0.4, 0.5 & 0.75 µM)^74^. Our prior analysis however, did not account for potential differences in dynamic structural changes. For instance, partial or total retraction of a secondary neurite could be accompanied by elongation of a new secondary neurite at a different location within the same cell. Different examples of dynamic structural rearrangements are shown in Fig. 1. Another example of these dynamic structural changes taking place during early development would be a partial or total retraction of a primary neurite occurring in parallel to elongation of a new primary neurite at a different site within the soma. As mentioned earlier, here we studied these dynamic structural changes by assigning a score to each structural modification as depicted in Table 3. The more drastic the structural rearrangement, the higher the score (see methods section). The sum of all structural changes or SDI was obtained only in those individuals in which we had pictures of MDNCs at T0hr and T1hr (Table 2). Two of our patients were not taking any medications (Table 2). Therefore, our analysis includes comparisons between CTL versus either all patients with SCZ or only medicated patients (MED). Contrasting unmedicated patients (UNMED) with the other three groups was only statistically valid for MDNCs cultured under control conditions, while comparisons between SCZ, CTL and MED were all statistically sound after treatments with colchicine 0.4, 0.5 and 0.75 µM. The number of individuals as well as the number of MDNCs included in each statistical analysis are described in Table 2.

Multilevel model results showed that in the colchicine 0.4 µM condition, SDI was significantly higher in SCZ vs. CTL (*b* = 5.27, 95% CI [0.64, 10.05]; Fig. 2F). In the control, colchicine 0.5 µM (Fig. 2G), and colchicine 0.75 µM conditions, there were no differences in SDI between groups (control: *b* = −1.90, 95% CI [−5.05, 1.16]; colchicine 0.5 µM: *b* = 1.76, 95% CI [−0.61, 4.15]; colchicine 0.75 µM: *b* = 1.23, 95% CI [−1.98, 4.38]). To estimate effect sizes, we calculated Hedge’s *g*, with SDI averaged within sample, and sample SDI averaged within participants. The effect sizes were large comparing cells between SCZ and CTL after treatments with colchicine 0.4 and 0.5 µM (Table 6).

**Table 6:** Descriptive statistics showing effect sizes between-group differences in SDI.

	Group						
	CTL	SCZ	MED	UNMED	CTL vs. SCZ	CTL vs. MED	CTL vs. UNMED
Condition	*M* (*SD*)	*M* (*SD*)	*M* (*SD*)	*M* (*SD*)	*g*	*g*	*g*
Control	13.96 (2.58)	11.98 (3.79)	11.24 (3.86)	14.96 (1.82)	0.57	0.78	0.36
Colchicine 0.4 µM	7.56 (2.88)	12.87 (4.75)	12.08 (4.85)	–	1.16	0.97	–
Colchicine 0.5 µM	4.40 (1.23)	6.42 (2.59)	6.43 (2.75)	–	0.82	0.78	–
Colchicine 0.75 µM	5.09 (2.22)	6.35 (2.50)	5.79 (1.98)	–	0.47	0.31	–

*M* mean, *S* standard deviation, *CTL* controls, *SCZ* patients with schizophrenia, *MED* medicated patients with SCZ, *UNMED* unmedicated patients with SCZ. Dashes mean that there was only one participant in the SCZ-unmedicated group and therefore the CTL vs. SCZ unmedicated comparisons could not be performed. *g* = Hedge’s *g*.

Since these results suggest MDNCs from SCZ responded differently to colchicine 0.4 µM than cells from CTL, we performed a 2 (group) $\times$ 4 (condition) ANOVA, with cells nested within samples and samples nested within participants. Contrasts’ 95% CIs were estimated with *n* = 1000 bootstrap sampling. Contrasts showed that MDNCs cultured under control conditions from CTL showed statistically significant differences when compared to each colchicine dose (Table 7). While MDNCs from SCZ evidenced no difference when cells cultured under control conditions were compared to cells treated with colchicine 0.4 µM (Table 7). Moreover, MDNCs from CTL evidenced no statistical differences among the three colchicine concentrations, since all concentrations led to a drastic decrease in SDI. Whereas cells from SCZ, demonstrated statistically significant differences when contrasting colchicine 0.4 µM versus either colchicine 0.5 or 0.75 µM (Table 7). All these analyses were done blinded to diagnosis and cell treatment.

**Table 7:** Bootstrapped pairwise comparison results from multilevel models comparing SDI by group (CTL vs. SCZ) and condition.

Condition	CTL		SCZ	
	*est*	*95% CI*	*est*	*95% CI*
Control vs. colchicine 0.4	6.46	3.03, 9.74	−0.61	−3.21, 1.95
Control vs. colchicine 0.5	9.62	6.47, 12.78	5.48	3.22, 7.71
Control vs. colchicine 0.75	8.59	5.06, 12.20	5.46	3.14, 7.80
Colchicine 0.4 vs. colchicine 0.5	3.17	−0.61, 6.82	6.09	3.60, 8.58
Colchicine 0.4 vs. colchicine 0.75	2.14	−2.12, 6.34	6.06	3.40, 8.80
Colchicine 0.5 vs. colchicine 0.75	−1.03	−4.75, 2.76	−0.03	−2.23, 2.45

*CTL* controls; *SCZ* schizophrenia.

### Haloperidol and clozapine effects on structural dynamic changes in MDNCs

**Fig. 3:**
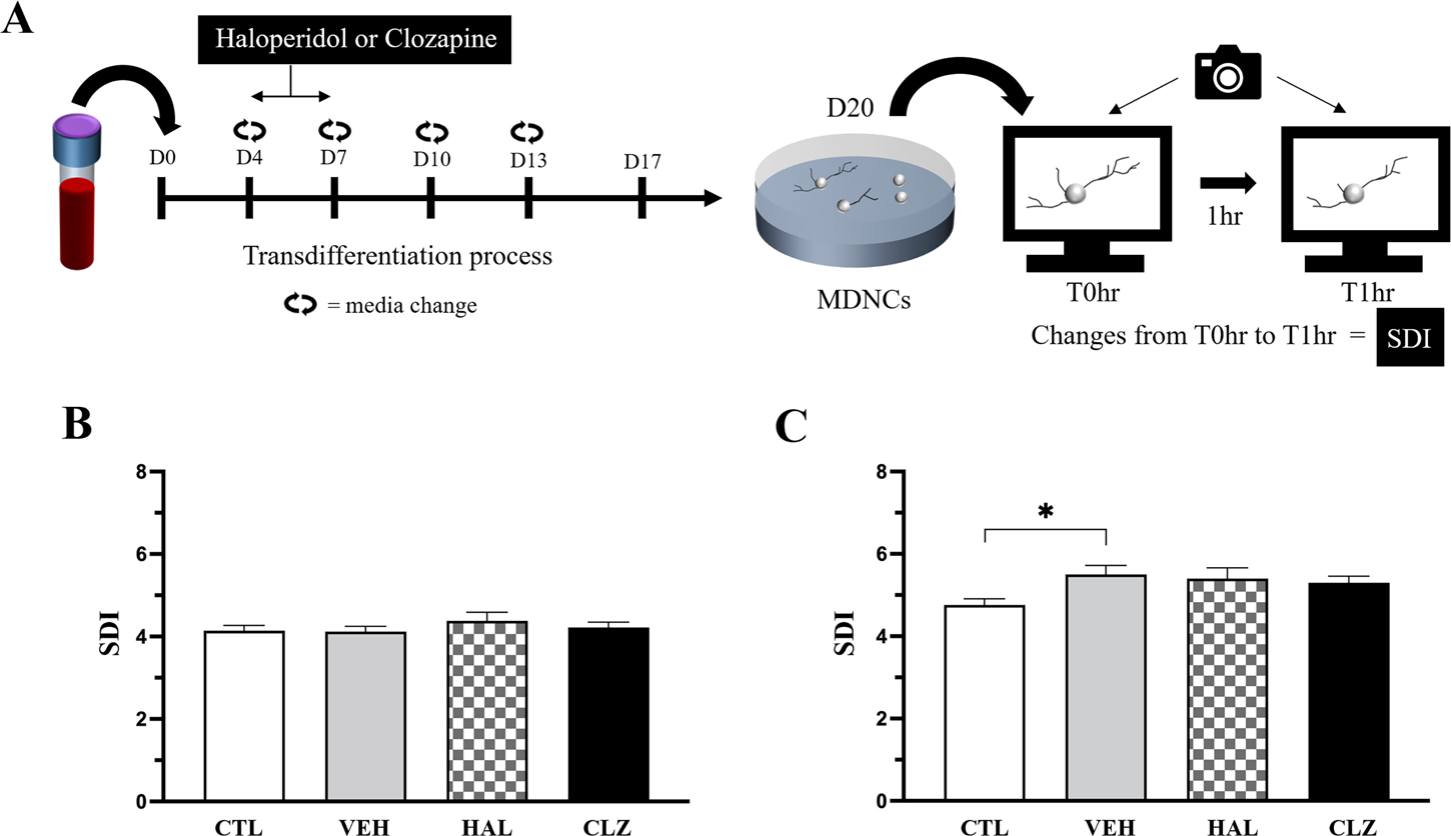
Effects of antipsychotics on Structural Dynamic changes in MDNCs. **A** Diagram representing our transdifferentiation protocol starting from a blood sample and progressing from day 0 (D0) to day 20 (D20). Either haloperidol or clozapine were added on day 4 (D4) and left until day 7 (D7). Also shown are schematics of how pictures of MDNCs were taken in order to determine SDI. **B** Bar graphs comparing SDI of MDNCs treated from D4 to D7 with either vehicle (VEH; *n* = 206), haloperidol (HAL; *n* = 114), clozapine (CLZ; *n* = 177) or under control conditions (CTL; *n* = 205). **C** Bar graphs comparing SDI of MDNCs treated from D4 to D7 with either VEH (*n* = 150), HAL (*n* = 104), CLZ (*n* = 165) or CTL (*n* = 183) and then exposed to colchicine 0.4 µM on D20, *p = 0.023. Data is presented as mean ± SEM.

Since most patients in our cohort were taking antipsychotics, we investigated whether haloperidol and/or clozapine elicit structural dynamic changes in CTL MDNCs similar to those encountered in MDNDs from SCZ. Human monocytes remain in circulation for 2 to 3 days^99^ which is the time they are exposed to antipsychotics. To mimic these conditions, we exposed monocytes to circulating concentrations of haloperidol (20 ng/ml) and clozapine (500 ng/ml) during days 4 to 7 of the transdifferentiation process (Fig. 3A). A one-way ANOVA analysis indicated that neither haloperidol (HAL), nor clozapine (CLZ) or vehicle (VEH) impacted SDI in MDNCs when these cells were cultured under control conditions [F(3, 698) = 0.678, *p* = 0.565] (Fig. 3B). However, when MDNCs were treated with colchicine 0.4 µM, there were statistical differences [F(3, 598) = 3.63, *p* = 0.012]. Tukey post hoc comparisons showed that MDNCs treated with VEH had a statistically higher SDI than CTL cells (CTL, 4.76 ± 0.15; VEH, 5.57 ± 0.22; *p* = 0.023). HAL (5.44 ± 0.26) and CLZ (5.3 ± 0.16) were not statistically different from CTL; nonetheless, their SDIs were closer to VEH than to CTL (Fig. 3C). In fact, cells treated with HAL evidenced a statistical trend when compared to CTL (CTL, 4.76 ± 0.15; HAL, 5.44 ± 0.26; *p* = 0.077). MDNCs included in these analyses came from four healthy individuals with one individual giving blood twice. These analyses were also done blinded.

## Discussion

Studying SCZ at a cellular level remains difficult in part because access to developing neurons coming directly from patients is limited. To overcome this challenge, several cellular approaches have been created including Induced Pluripotent Stem Cells and transdifferentiation of somatic cells. We recently compared all cellular approaches available to study psychiatric and neurological illnesses^85^ and among the fastest and more practical methodologies to deliver neuronal-like cells directly from patients are MDNCs. In contrast with other cellular approaches^80–85^, MDNCs have shown reproducible results in serial samples from healthy individuals^74,78^. A disadvantage of working with MDNCs came from its uncertain location along the neurodevelopmental timeline. Our earlier publications indicated that despite MDNCs conduction of electrical activity, these cells are not differentiated into any particular neuronal type^78^, suggesting MDNCs resemble neurons at very early stages of development^78^. We had also previously shown that specific monocyte markers like the surface protein CD14 as well as genes such as *ITGAM* and *CCR2* were either, no longer present or considerably decreased in MDNCs^78,79^. These prior results are in line with our current data. Here, through the use of scmap^94^ and signature genes stratifying human cortical neurons at different stages of development^98^, we were able to cluster MDNCs with human NPCs (Table 4). At the same time, MDNCs are no longer clustered with monocytes (Table 5 & Supplementary Table 6). Moreover, the similarities between MDNCs and NPCs extend to their structural plasticity (Fig. 2E). The structure of MDNCs and human NPCs is highly dynamic, more so than that of HDNs (Fig. 2A, E). Therefore, two different lines of evidence now locate MDNCs together with human NPCs in the neurodevelopmental timeline.

The high degree of structural plasticity present in MDNCs and its resemblance with NPCs, make these cells a suitable approach to study potential deficits in neurostructural dynamics carried by patients with SCZ. The resemblance with NPCs is significant because previous indirect evidence from humans^96,97^ and in vivo data from rodents^19,31^ together with our current comparison between NPCs and HDNs (Fig. 2A, E), indicate that as neurodevelopment progresses neurostructural plasticity diminishes. Since neurostructural rearrangements are more drastic in very early stages of neurodevelopment, then potential deficits in neurostructural modifications would be easier to detect in cells such as NPCs or MDNCs as opposed to HDNs. In HDNs as well as in mature adult neurons, changes in the cellular structure are more subtle and thus more difficult to capture. Given that the stage of neuronal differentiation directly impacts the degree of structural plasticity, the level of neuronal differentiation must be comparable between cohorts to prevent it from becoming a confounder when studying dynamic transformations in the neuronal shape. This is particularly relevant for MDNCs, as we previously reported that patients with SCZ evidenced a higher percentage of differentiated cells than CTL^74^. Here we analyzed more than 10,000 cells (Table 1) and our results show that the number of differentiated cells remains constant between groups (Table 1). Therefore, it was the number of undifferentiated cells and not the number of MDNCs, which led to the gap between SCZ and CTL in percentage of differentiated cells we previously reported^74^. Nonetheless, the possibility that MDNCs from patients with SCZ are more differentiated still exists, as we showed in a prior publication, these cells expressed higher levels of nestin than MDNCs from CTL^74^. A more advanced level of maturity in MDNCs from individuals with SCZ should have revealed differences in SDI after comparisons with CTL cells under control cultured conditions (in the absence of colchicine). However, no differences in SDI were found (Table 6). If MDNCs from patients were in fact more differentiated than cells from CTL, our current results would indicate that the neurostructure of MDNCs from patients is hyperdynamic, since the expected drop in structural dynamism that accompanies advances in neuronal differentiation did not occur. While this later statement is just speculative, our results with colchicine 0.4 µM showed that the neurostructure of MDNCs from patients with SCZ is hyperdynamic at least under certain circumstances (Fig. 2F). Dynamic changes in the structure of MDNCs from patients were not halted by the lowest concentration of colchicine tested (Fig. 2F). Instead, SDI remained unaffected by colchicine 0.4 µM (Fig. 2F & Table 7). Similarly, when within groups differences were assessed, MDNCs from patients with SCZ treated with colchicine 0.4 µM were not statistically different from SCZ cells cultured under control conditions (Table 7). The neurostructure of MDNCs from patients with SCZ remained dynamic regardless of whether they were treated or not with colchicine 0.4 µM.

Higher concentrations of colchicine (0.5 and 0.75 µM) did stop MDNCs’ structural dynamism in either SCZ or CTL (Table 7). It is possible that the differences we observed between the lowest versus higher concentrations of colchicine could be driven by this compound’s mechanism of action, since low concentrations of colchicine form a tubulin-colchicine complex that arrests growth of microtubules^38–41^ and thus, keeps the neuronal shape static^38^. While at higher concentrations, colchicine elicits microtubule depolymerization leading to neurite retraction^41–44^.

Differences in the number and length of neuronal extensions could also be a potential confounder. In a prior publication, we showed that MDNCs from SCZ had longer secondary neurites and more primary neurites^74^. However, if neuropil size would have influenced our results, we would have observed such differences in group comparisons at baseline, when MDNCs were cultured under control conditions and not exclusively by the lowest concentration of colchicine tested. On the other hand, pruning of neuronal extensions elicited by colchicine cannot be a factor in our current results, since we previously showed that the level of neurite retraction in MDNCs was similar between SCZ and CTL independently of the concentration of colchicine used^74^.

Postmortem studies and ONCs in vitro have shown abnormal distribution and less microtubules in neurons of patients with SCZ^49,56–58^ (Supplementary Table 1). But whether these microtubules were properly functioning was not assessed until Brown and colleagues reported that microtubules in olfactory neuroepithelial cells from patients with SCZ were resistant to the depolymerization effects of nocodazole^48^ (Supplementary Table 1). Our results support these findings. But we did not examine microtubules in isolation. Instead, we studied microtubules within the context of one of its most important functions; shaping the neuronal structure. We found that in MDNCs from patients with SCZ, colchicine 0.4 µM did not halt microtubule dynamics in charge of eliciting rapid neurostructural changes. Thus, there is now evidence from two independent teams, studying two separate cohorts and using two different cellular approaches, indicating that microtubule polymerization in neuronal-like cells from patients with SCZ is resistant to compounds such as nocodazole and colchicine at least under certain circumstances. What remains to be addressed is the potential influence of antipsychotics on these results.

Antipsychotics can affect the neuronal structure in vitro^100^ and preliminary evidence suggests antipsychotics elicit neurite outgrowth via microtubule enlargement^101^. But whether antipsychotics directly influence microtubule polymerization remains under study. A comparison via western blots between two unmedicated patients and four individuals with SCZ taking antipsychotics, revealed differences in the subcellular distribution of β-III-tubulin in olfactory neuronal precursors^102^. While medicated patients evidenced no changes in the amount of cytoskeletal and cytosolic fraction of β-III-tubulin when compared to CTL, unmedicated individuals with SCZ, presented lower levels of this protein in the cytosol^102^. It is difficult however, to draw any definite conclusions from such comparisons as neurons in the olfactory neuroepithelium modify the subcellular distribution of β-III-tubulin as development progresses^103^ and the comparisons between CTL, medicated and unmedicated patients with SCZ did not address potential variations in cell differentiation among groups^102^. Other investigators have directly addressed the impact of antipsychotics on microtubule polymerization. In fact, several studies conducted by independent teams have demonstrated that chlorpromazine at circulating concentrations, inhibits microtubule polymerization^104–106^. There is also evidence that thioridazine and trifluoperazine impact microtubules comparably to chlorpromazine^106^. Likewise, a recent report found that clozapine arrests microtubule polymerization in vitro by directly binding tubulin heterodimers^107^. All such studies suggest that at least some antipsychotics could potentially arrest a hyperdynamic neuronal structure. Consequently, our results could not be explained by the effects of antipsychotics. However, all experiments about antipsychotics’ effects on microtubule polymerization were conducted using either animal models or purified tubulin from cows or pigs^104–107^. Since these approaches do not carry the genetic susceptibility to SCZ, its translatability to MDNCs, olfactory neuronal precursors, or neurons in general, is yet to be determined.

We attempted to control for the potential confounding effects of medications by two means; recruiting unmedicated patients (Table 2), and treating MDNCs from healthy individuals with circulating concentrations of either clozapine or haloperidol (Fig. 3A–C). Unfortunately, both approaches were unsuccessful. Even though monocytes are exposed to antipsychotics only during the 2 to 3 days they remain in circulation^99^ and once isolated, we kept them in culture for over 20 days, changing media four times during the transdifferentiation process^78^ (Fig. 3A), these medications could still affect MDNCs, as evidenced by the effects of DMSO (Fig. 3C). We found that DMSO, which was used as vehicle to dissolve clozapine and haloperidol, increased SDI in MDNCs (Fig. 3C). In line with these unexpected results is a recent publication indicating that DMSO can increase tubulin polymerization^107^. Since our vehicle was active, we were unable to assess whether clozapine or haloperidol impact SDI in MDNCs. In similar fashion, we did not have enough unmedicated patients to conduct a sound statistical analysis (Table 2). Consequently, we cannot rule out the possibility that antipsychotics may have influenced our results.

Large genetic studies have encountered significant associations between microtubule-related genes and SCZ^54,55^. More targeted analyses have led to the identification of genetic anomalies that impact neuron-specific post-translational modifications of microtubule proteins^108^. Such post-translational modifications modulate microtubule development and dynamics^108^. A single-nucleotide polymorphism study found that a variant of the *MAP6* gene was more prevalent in patients with SCZ than in controls^109^. This association resulted in increased mRNA expression of *MAP6* in the dorsolateral prefrontal cortex of affected individuals^109^. Interestingly, MAP6 sometimes identified as STOP, is capable of stabilizing microtubules^110^. It is also well known that a balanced translocation affecting the *DISC1* gene segregates highly with SCZ^111–114^. While the role of DISC1 protein is still under investigation, several studies indicate that DISC1 regulates microtubule-dynamics and when *DISC1* expression is altered, the neuronal structure is compromised^115^. Given that all these genetic studies point to a relationship between microtubule-related genes, SCZ and deficits in the neuronal structure, it would have been logical to pursue a correlation between our single-cell RNA sequencing results and the SDI data we obtained. But our experimental design did not allow for such correlation. We do not have single-cell RNA sequencing data from those individuals in which we analyzed the MDNCs structure. Nonetheless, we firmly believe that the pursue of experiments linking microtubule-related genes, SCZ and anomalies in the neuronal structure is imperative, given its potential contribution in unveiling this disorder’s pathophysiology.

Another important point to consider is that the neuronal shape is also dependent on the actin cytoskeleton. The actin cytoskeleton, which organizes in microfilaments, works in tandem with microtubules to transform the neuronal structure in response to environmental needs, particularly during neurodevelopment^14,15^. Just as with microtubules, the actin cytoskeleton has been implicated in the pathophysiology of SCZ. Postmortem analyses have shown that actin polymerization is reduced in the anterior cingulate cortex of patients with SCZ^116^. In support of this finding, are studies linking mutations in genes involved in actin polymerization to SCZ^117^. Moreover, results obtained from human IPSCs and supported by experiments with rodents, indicate that proteins involved in regulating actin dynamics are compromised in SCZ^118^. Therefore, assessing dynamic changes in the neuronal structure has to, not only involve microtubules, but also consider microfilaments, something that we fail to do in this study.

There are several limitations that have to be considered when interpreting our results. While MDNCs mimic dynamic neurostructural changes found in human NPCs, MDNCs are not neurons. In addition, our results come from two-dimensional assessments of neuronal-like cells, future studies have to be performed using three-dimensional approaches which more closely resemble the neuronal environment. Also important to consider is that our single-cell RNA sequencing results come from only 17 MDNCs and there is variability in gene expression between cells. Further experiments, including a higher number of MDNCs are needed to draw more solid conclusions. Finally, as we previously emphasized, the potential confounding effects of antipsychotics cannot be excluded.

## Conclusions

Here we present for the first time, results coming directly from live human neurons, supporting previous indirect evidence from postmortem human studies^96,97^ and in vivo data from rodents^19,31^, pointing to a decrease in neurostructural plasticity as neurodevelopment progresses. In addition, our results indicate patients with SCZ present hyperdynamic microtubule-based structural changes in MDNCs after treatment with colchicine 0.4 µM. We therefore linked, at least under some circumstances, deficits in microtubules dynamics with anomalies in the neuronal shape in SCZ. Considering that MDNCs genetically resemble human NPCs and that both cell types have comparable structural plasticity, then it is possible that under certain conditions, NPCs from patients with SCZ are structurally hyperdynamic. In support of this theory are inconsistencies found when studying snap shots of the neuronal structure, like in postmortem analyses, which in the case of SCZ, have evidenced decreased^63–67^, increased^68,69^ or unchanged^119,120^ neuronal extensions. For firm conclusions to be established however, further research directly in patients’ cells involving microtubule polymerization/depolymerization within the context of dynamic neurostructural changes that incorporates modifications in the level of expression and phosphorylation of microtubule-associated protein-2 (MAP2) are warranted. Integrating the study of MAP2 is relevant because this protein plays a prominent role in transforming the neuronal shape^14^ and, in addition, it has been repeatedly associated with SCZ despite delivering inconsistent results, like increased^121^, decreased^122,123^ or unchanged expression^124^ in the same brain regions. Perhaps studying MAP2 within the framework of constant rearrangements in the neuronal shape will clarify those inconsistencies. Also paramount to establish whether dynamic neurostructural modifications are defective in SCZ, is to eliminate the potential confounding effects of medications. To this end, it will be necessary, not only to test the effects of antipsychotics in cells from healthy individuals but also challenge cells from patients with SCZ.

## Supplementary information

Supplemental Tables & Figures

Supplemental Table 3

Supplemental Table 5

## Data Availability

All data generated during this study are available upon request. Our data is not yet publicly available because we are still analyzing it for future publications.
