# Supplementary material for: Hyperdynamic microtubule-based structural changes in Monocyte-Derived-Neuronal-like cells from patients with schizophrenia
Source: Schizophrenia (Heidelb). 2026 Apr 30;12(1):60. doi: 10.1038/s41537-026-00751-0 (PMC13338171; doi:10.1038/s41537-026-00751-0)
Supplement: Supplementary file 1 — Supplemental Tables & Figures [file 41537_2026_751_MOESM1_ESM.pdf]

Supplementary Table 1. Findings on microtubules and microtubular proteins in patients with schizophrenia.

Reference	Methodological Approach	Tissue/Cells	Main Results
Pavlova, 1981	Electron microscopy	Brains of human embryos	Higher number of microtubules. Microtubules shape was convoluted and its normal orientation was lost.
Uranova, 1988	Electron microscopy	Postmortem brain tissue from prefrontal cortex	Reduced number or absent microtubules in dendrites. Microtubules were swollen.
Uranova & Aganova, 1989	Electron microscopy	Postmortem brain tissue from anterior limbic cortex	Lack of microtubules in synapses.
Oifa & Uranova, 1991	Electron microscopy	Postmortem brain tissue from prefrontal cortex	Absence of microtubules in dendrites.
Prabakaran et al, 2004	Proteomics	Postmortem brain tissue from prefrontal cortex	Decreased expression of α-tubulin.
Bauer et al, 2009	Western blot	Postmortem brain tissue	No changes in the expression of β-tubulin in any of the four brain areas studied.*
English et al, 2009	Proteomics	Postmortem brain tissue from DLPFC	Decreased expression of β-tubulin.
Moehle at al, 2012	Western blot	Postmortem brain tissue	Increased expression of β1-tubulin in DLPFC. Decreased expression of β1-tubulin in ACC. No change in hippocampus or superior temporal gyrus.
Solís-Chagoyán et al, 2013	Immunofluorescence with anti-β-III-tubulin	Neuronal Precursors obtained via exfoliates of nasal mucosa	Wider unstained areas of β-III-tubulin in the cytoplasm of SCZ cells.
Brown et al, 2014	Cultured cells were treated with 10μM nocodazole	Olfactory Neuroepithelial Cells (ONCs)	ONCs from SCZ were resistant to depolymerizing effects of nocodazole.

*Anterior cingulate cortex (ACC), dorsolateral prefrontal cortex (DLPFC), hippocampus and primary visual cortex.

Supplementary Table 2. Number of MDNCs per subject for each experiment comparing patients versus controls.

Subject/ Patient	Total* MDNCs	Control Conditions	Colchicine 0.4µM	Colchicine 0.5µM	Colchicine 0.75µM
Subject 1	169				
Subject 2	740	89	140	163	120
Subject 3	207	59			
Subject 4	134	20	16		
Subject 5**	371				
Subject 6	286	77			
Subject 7**	301	83		113	82
Subject 8	242				
Subject 9	369	90		82	94
Subject 10	243	24			
Subject 11	306	23	45	39	
Subject 12	256				
Subject 13	309		65		
Patient 1	383	64	41	53	68
Patient 2	696	172		156	176
Patient 3	835			103	123
Patient 4+	634			154	
Patient 5	321				72
Patient 6	646	168	73	125	111
Patient 7 ++Unmed	328	113			
Patient 8	247	38	55	43	
Patient 9	419	80	61	68	50
Patient 10+	536	83	107	94	128
Patient 11	301	67		71	52
Patient 12	218	60	26	29	
Patient 13 ++Unmed	215	34	16	36	29
Patient 14	365				

*Includes all MDNCs obtained from each patient. **Same individual tested twice.
+Same patient tested twice. ++Unmedicated patient.

Supplementary Table 6. Characterization via scmap of MDNCs based on 100 monocyte specific genes.

MDNC	CLUSTER	SCORE
1	unassigned	0.284137308
2	unassigned	0.268117753
3	unassigned	0.243851769
4	unassigned	0.152736283
5	unassigned	0.346010178
6	unassigned	0.245479073
7	unassigned	0.230174507
8	unassigned	0.203938406
9	unassigned	0.257526339
10	unassigned	0.240490149
11	unassigned	0.188731017
12	unassigned	0.340659034
13	unassigned	0.161585158
14	unassigned	0.294455276
15	unassigned	0.061799889
16	unassigned	0.177118883
17	unassigned	0.295481548

Supplementary Table 4. Characterization via scmap of MDNCs based on 100 Human Developing Cortex genes.

MDNC	CLUSTER	SCORE
1	NPC	0.781249368
2	NPC	0.765195375
3	NPC	0.786843835
4	unassigned	0.554099815
5	NPC	0.795855204
6	NPC	0.710475812
7	NPC	0.748023125
8	NPC	0.813830938
9	NPC	0.790085248
10	NPC	0.703074204
11	NPC	0.767801145
12	unassigned	0.204775955
13	unassigned	0.640632689
14	NPC	0.831931344
15	NPC	0.815762865
16	NPC	0.702083196
17	NPC	0.77278332

*Neuroprogenitor cell, **Gestation week 21

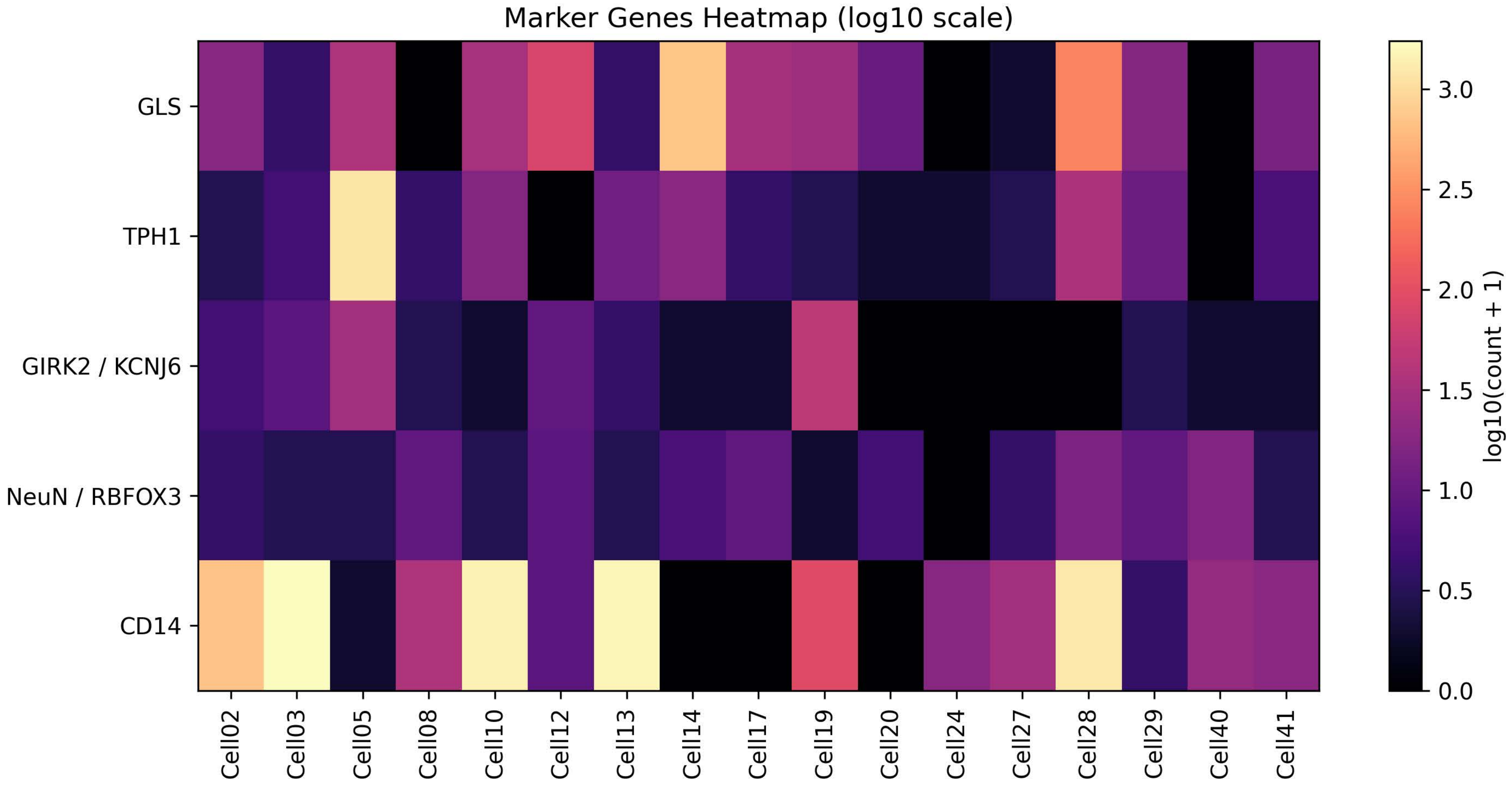

Marker Genes Heatmap (log10 scale)
GLS
TPH1
GIRK2 / KCNJ6
NeuN / RBFOX3
CD14
Cell02
Cell03
Cell05
Cell08
Cell10
Cell12
Cell13
Cell14
Cell17
Cell19
Cell20
Cell24
Cell27
Cell28
Cell29
Cell40
Cell41
log10(count + 1)
0.0
0.5
1.0
1.5
2.0
2.5
3.0

Supplementary Figure 1. Heatmap using representative marker genes across 17 MDNCs showing expected cell to cell variability in gene expression no unusually low gene detection.

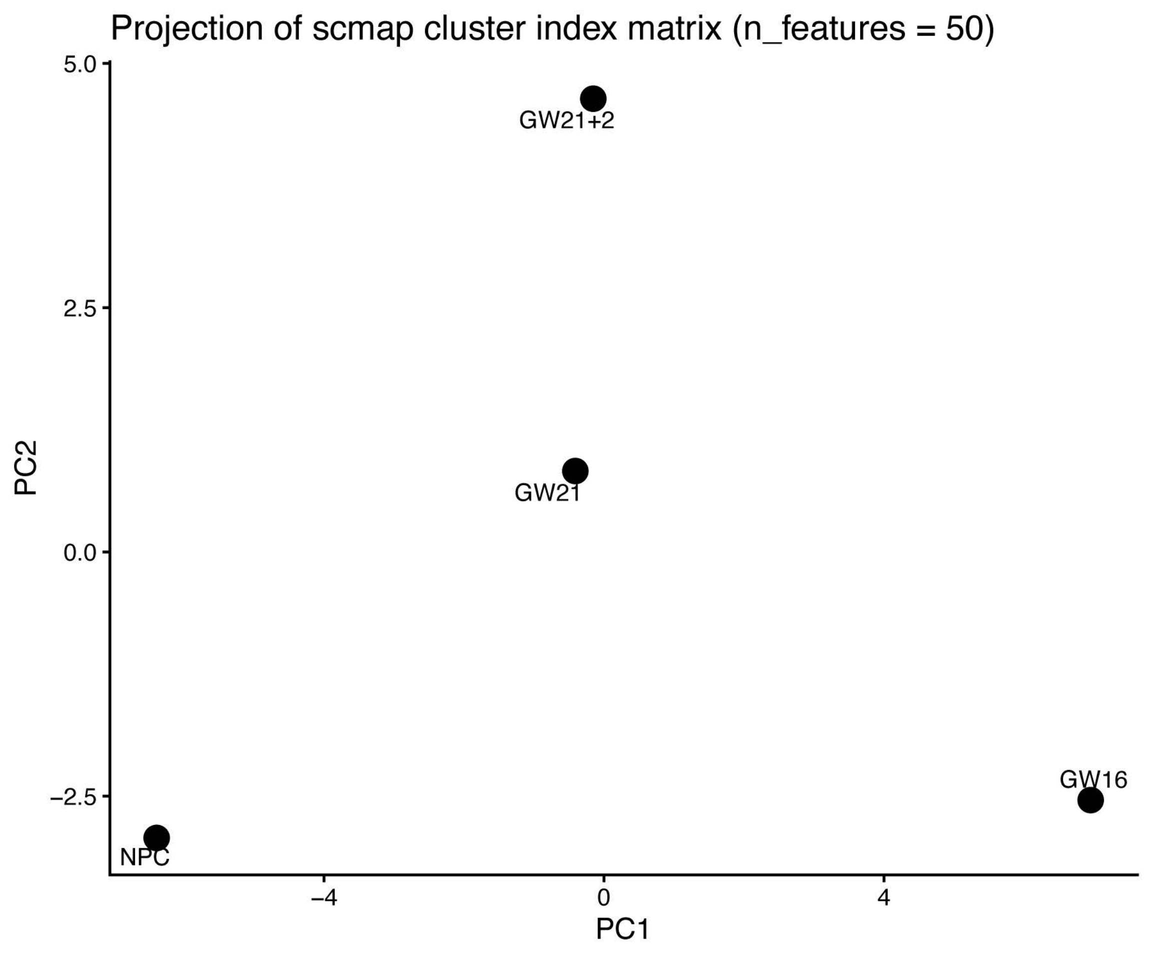

Projection of scmap cluster index matrix (n_features = 50)
5.0
2.5
0.0
−2.5
PC2
−4
0
4
PC1
GW21+2
GW21
GW16
NPC

Supplementary Figure 2. Projection of scmap cluster index matrix.

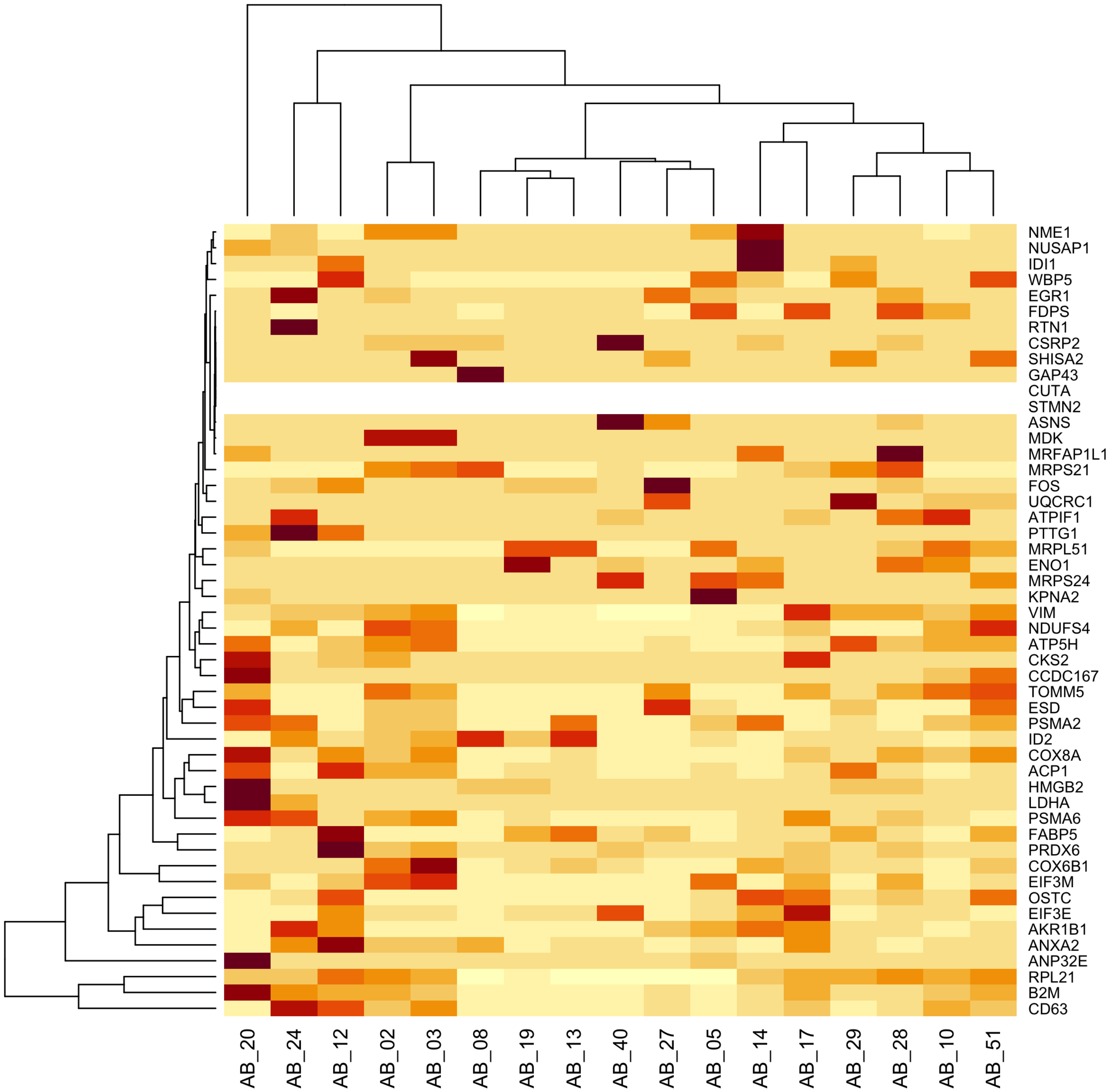
NME1
NUSAP1
IDI1
WBP5
EGR1
FDPS
RTN1
CSRP2
SHISA2
GAP43
CUTA
STMN2
ASNS
MDK
MRFAP1L1
MRPS21
FOS
UQCRC1
ATPIF1
PTTG1
MRPL51
ENO1
MRPS24
KPNA2
VIM
NDUFS4
ATP5H
CKS2
CCDC167
TOMM5
ESD
PSMA2
ID2
COX8A
ACP1
HMGB2
LDHA
PSMA6
FABP5
PRDX6
COX6B1
EIF3M
OSTC
EIF3E
AKR1B1
ANXA2
ANP32E
RPL21
B2M
CD63
AB_20
AB_24
AB_12
AB_02
AB_03
AB_08
AB_19
AB_13
AB_40
AB_27
AB_05
AB_14
AB_17
AB_29
AB_28
AB_10
AB_51

Supplementary Figure 3. Heatmap of 50 signature neuronal genes expressed in 17 MDNCs.

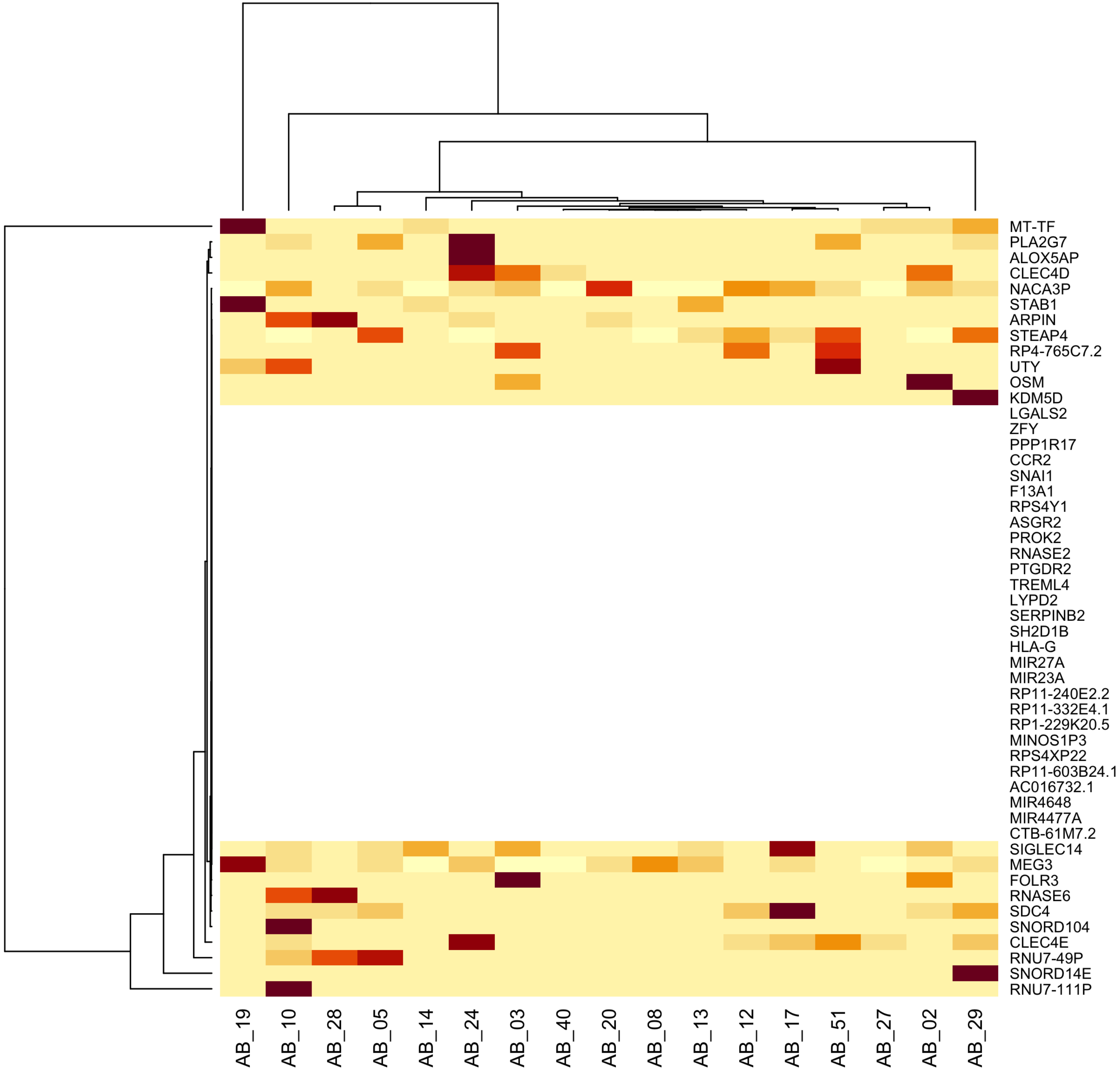
MT-TF
PLA2G7
ALOX5AP
CLEC4D
NACA3P
STAB1
ARPIN
STEAP4
RP4-765C7.2
UTY
OSM
KDM5D
LGALS2
ZFY
PPP1R17
CCR2
SNAI1
F13A1
RPS4Y1
ASGR2
PROK2
RNASE2
PTGDR2
TREML4
LYPD2
SERPINB2
SH2D1B
HLA-G
MIR27A
MIR23A
RP11-240E2.2
RP11-332E4.1
RP1-229K20.5
MINOS1P3
RPS4XP22
RP11-603B24.1
AC016732.1
MIR4648
MIR4477A
CTB-61M7.2
SIGLEC14
MEG3
FOLR3
RNASE6
SDC4
SNORD104
CLEC4E
RNU7-49P
SNORD14E
RNU7-111P
AB_19
AB_10
AB_28
AB_05
AB_14
AB_24
AB_03
AB_40
AB_20
AB_08
AB_13
AB_12
AB_17
AB_51
AB_27
AB_02
AB_29

Supplementary Figure 4. Heatmap of 50 signature monocytic genes expressed in 17 MDNCs.
